# Supplementary material for: Benzoquinoline Derivatives: An Attractive Approach to Newly Small Molecules with Anticancer Activity
Source: Int J Mol Sci. 2023 May 1;24(9):8124. doi: 10.3390/ijms24098124 (PMC10179047; doi:10.3390/ijms24098124)

International Journal of *Molecular Sciences*

MDPI

Supplementary Materials

Benzoquinoline Derivatives: An Attractive Approach to Newly Small Molecules with Anticancer Activity

Liliana Oniciuc [1], Dorina Amăriucăi-Mantu [1], Dumitrela Diaconu [1,2], Violeta Mangalagiu [2], Ramona Danac [1], Vasilichia Antoci [1] and Ionel I. Mangalagiu [1,2,*]

[1] Faculty of Chemistry, Alexandru Ioan Cuza University of Iasi, 11 Carol 1st Bvd, 700506 Iasi, Romania; lili_oniciuc@yahoo.com (L.O.); dorina.mantu@uaic.ro (D.A.-M.); dumitrela.diaconu@uaic.ro (D.D.); rdanac@uaic.ro (R.D.); vasilichia.antoci@uaic.ro (V.A.)

[2] Institute of Interdisciplinary Research-CERNESIM Center, Alexandru Ioan Cuza University of Iasi, 11 Carol I, 700506 Iasi, Romania; violeta.mangalagiu@uaic.ro

* Correspondence: ionelm@uaic.ro; Tel.: +40-232-201343

Content

1. NMR spectra of representative compounds**6b**, **6e**, **7b**, **7d**2-5
2. Anticancer results of all tested compounds **HybB[f]Q 6a–f** and **7a–e**5-9
3. CheckCIF files for X–ray data of compounds **6c** and **7c**10-17

1. NMR spectra of representative compounds**6b**, **6e**, **7b**, **7d**

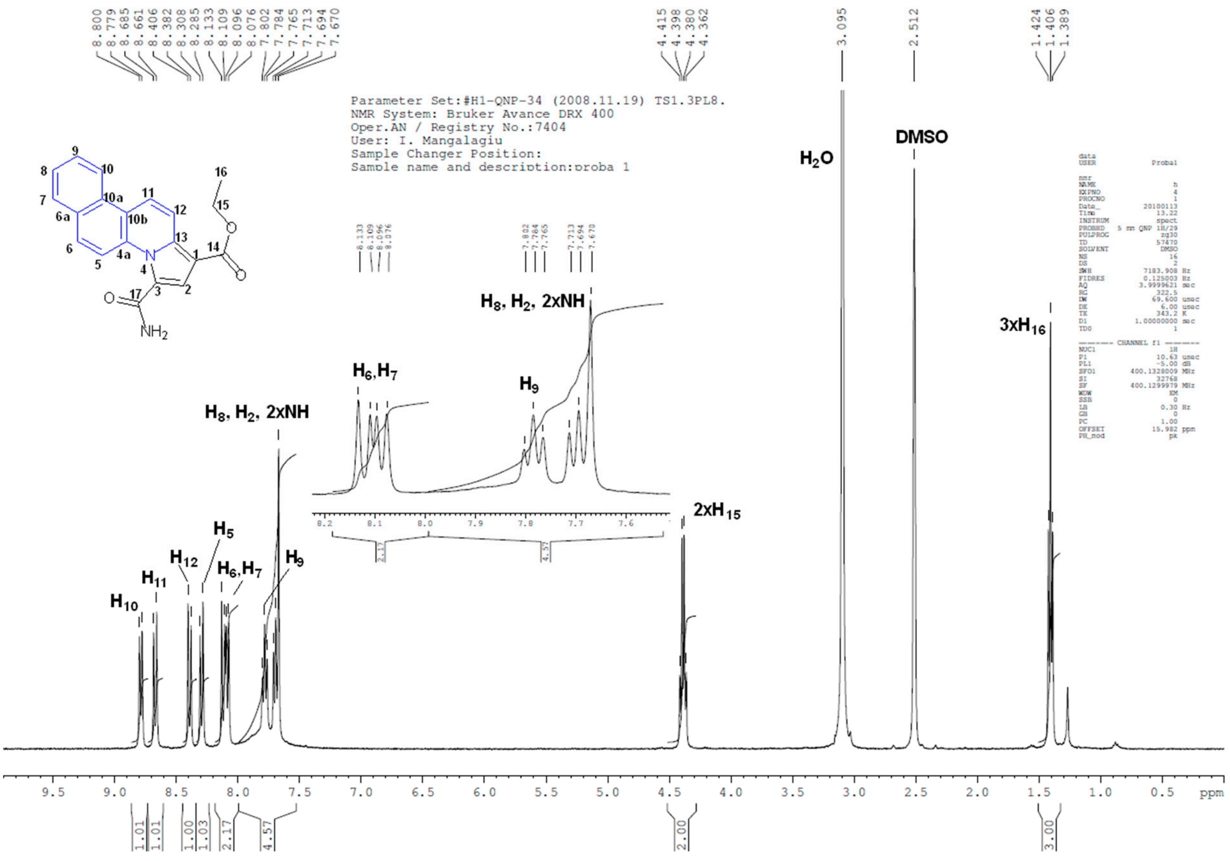

Figure S1. ^{1}H–NMR spectrum of ethyl 3–carbamoylbenzo[f]pyrrolo[1,2–a]quinoline–1–carboxylate (**6b**).

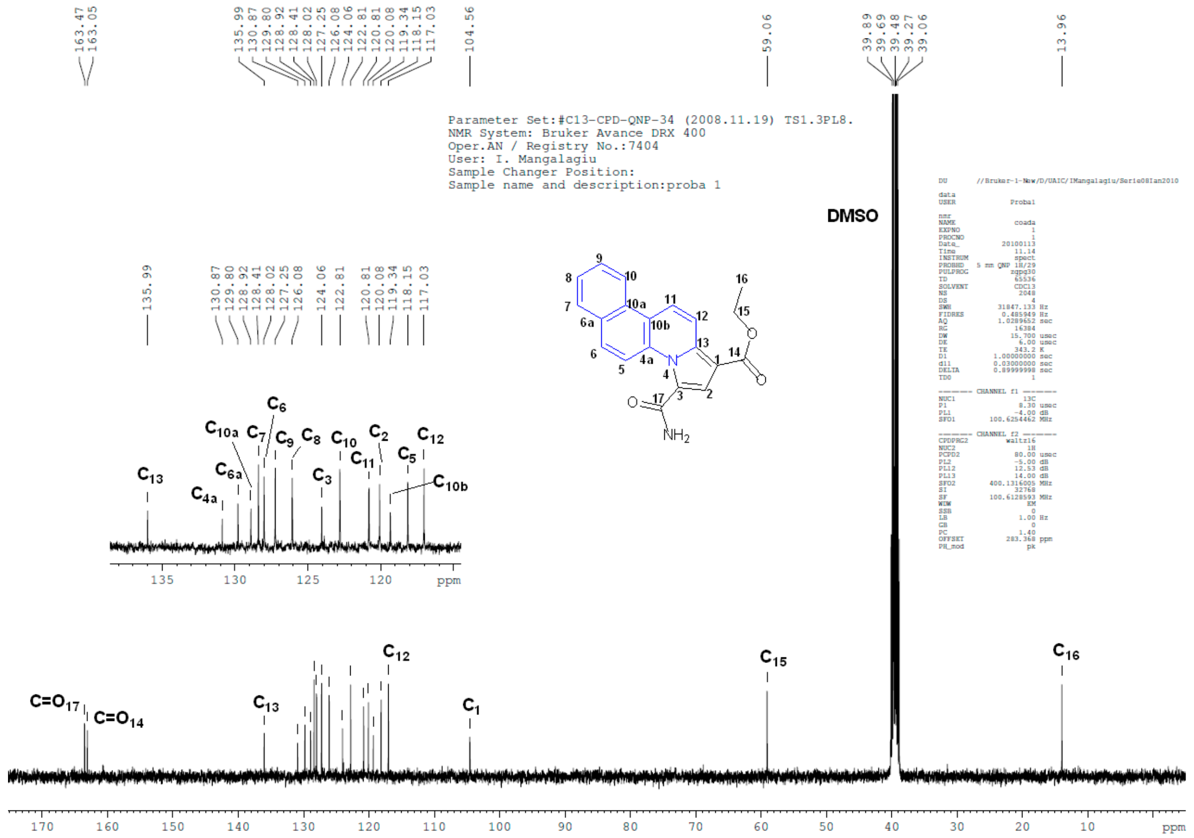

Figure S2. ^{13}C–NMR spectrum of ethyl 3–carbamoylbenzo[f]pyrrolo[1,2–a]quinoline–1–carboxylate (**6b**).

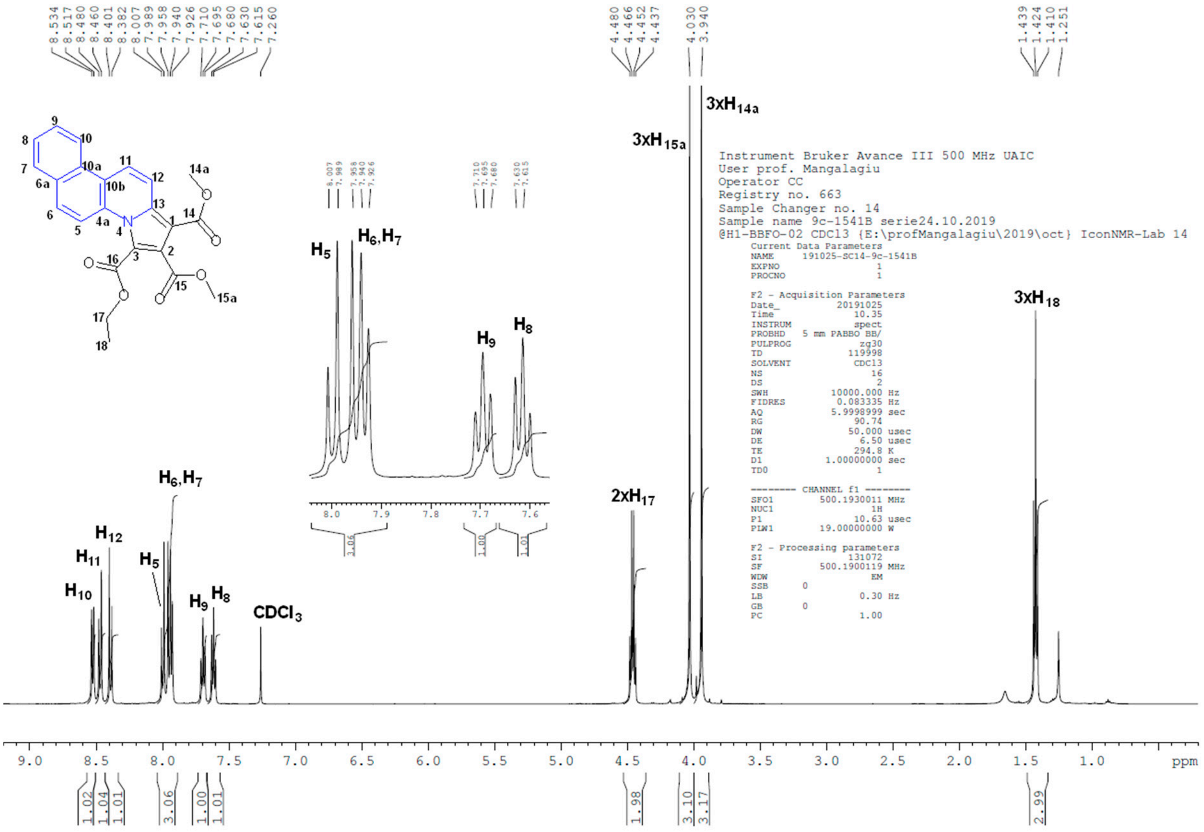

Figure S3. ^{1}H–NMR spectrum of 3–ethyl 1,2–dimethyl benzo[f]pyrrolo[1,2–a]quinoline–1,2,3–tricarboxylate (**6e**).

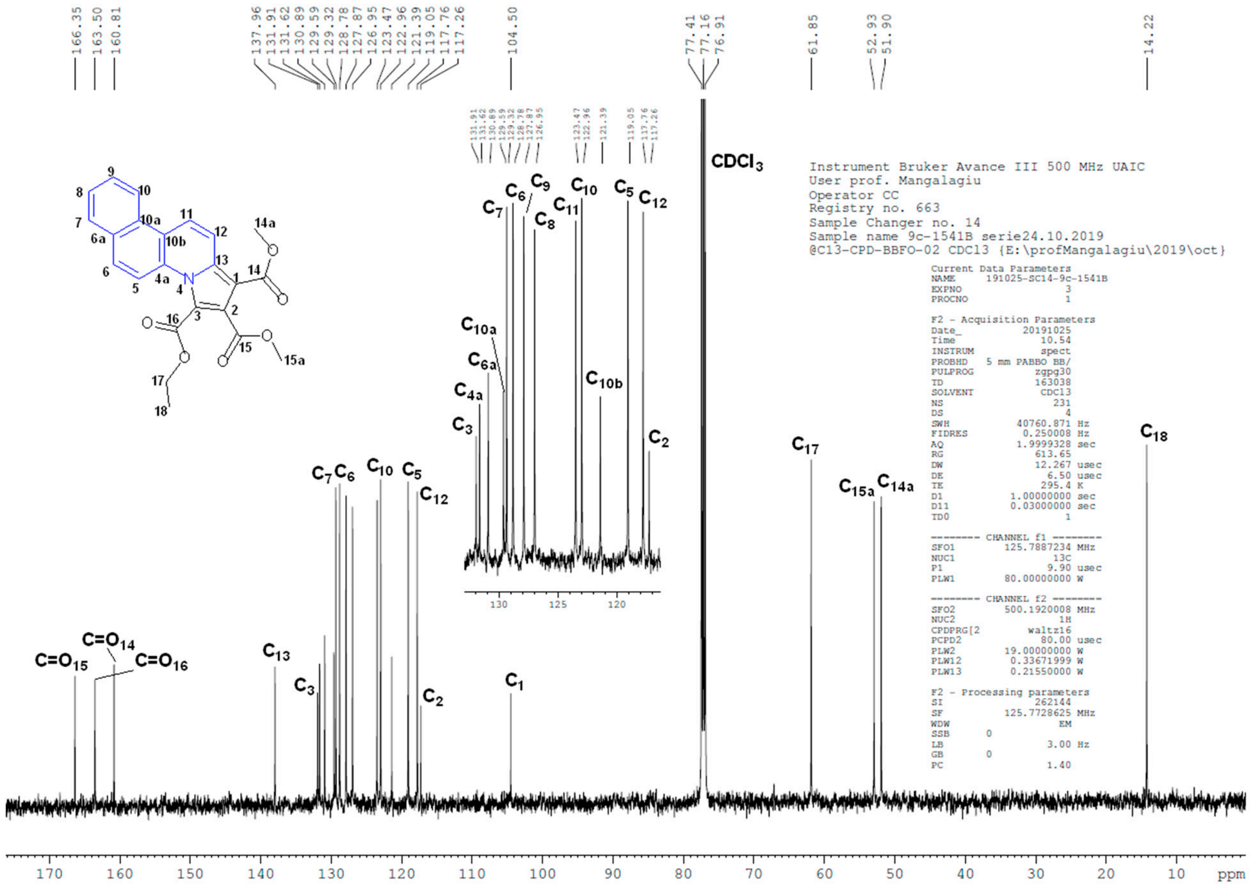

Figure S4. ^{13}C–NMR spectrum of 3–ethyl 1,2–dimethyl benzo[f]pyrrolo[1,2–a]quinoline–1,2,3–tricarboxylate (**6e**).

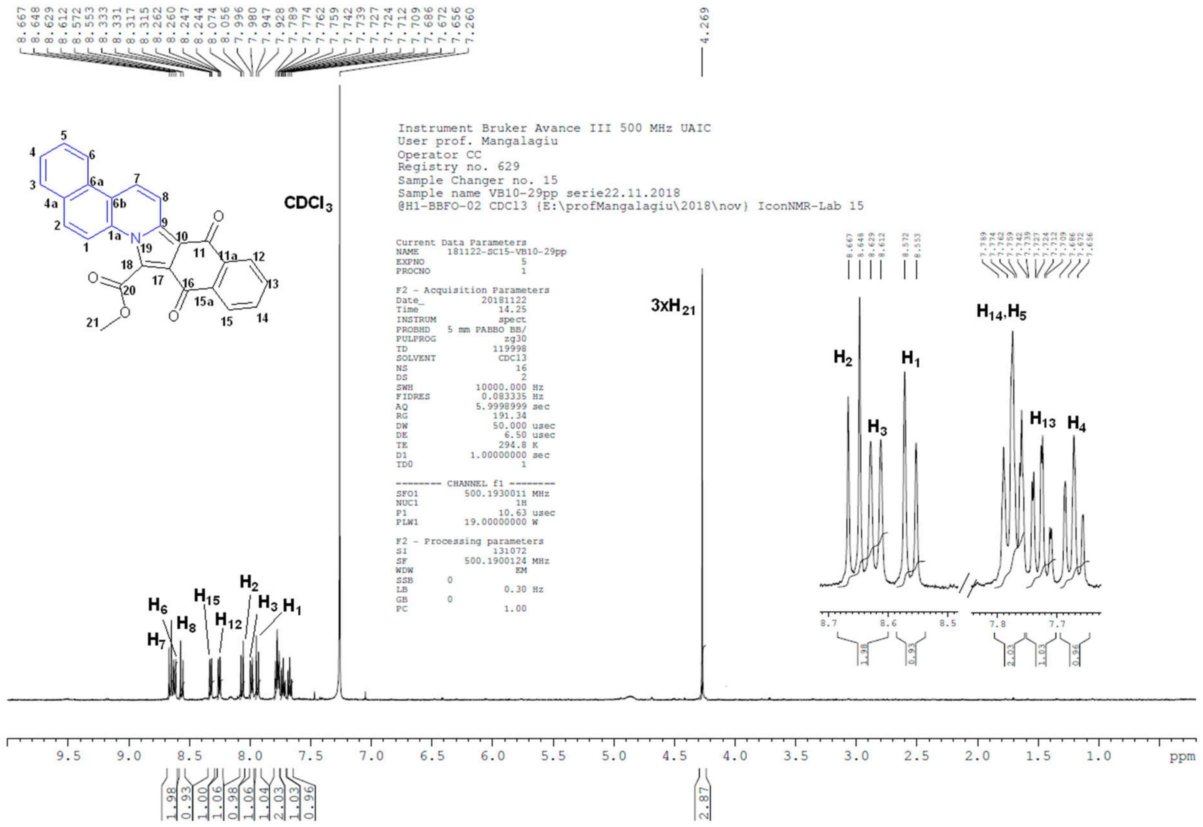

Figure S5. ^{1}H–NMR spectrum of methyl 9,14–dioxo–9,14–dihydrobenzo[f]benzo[5,6]isoindolo[2,1–a]quinoline–15–carboxylate (**7b**).

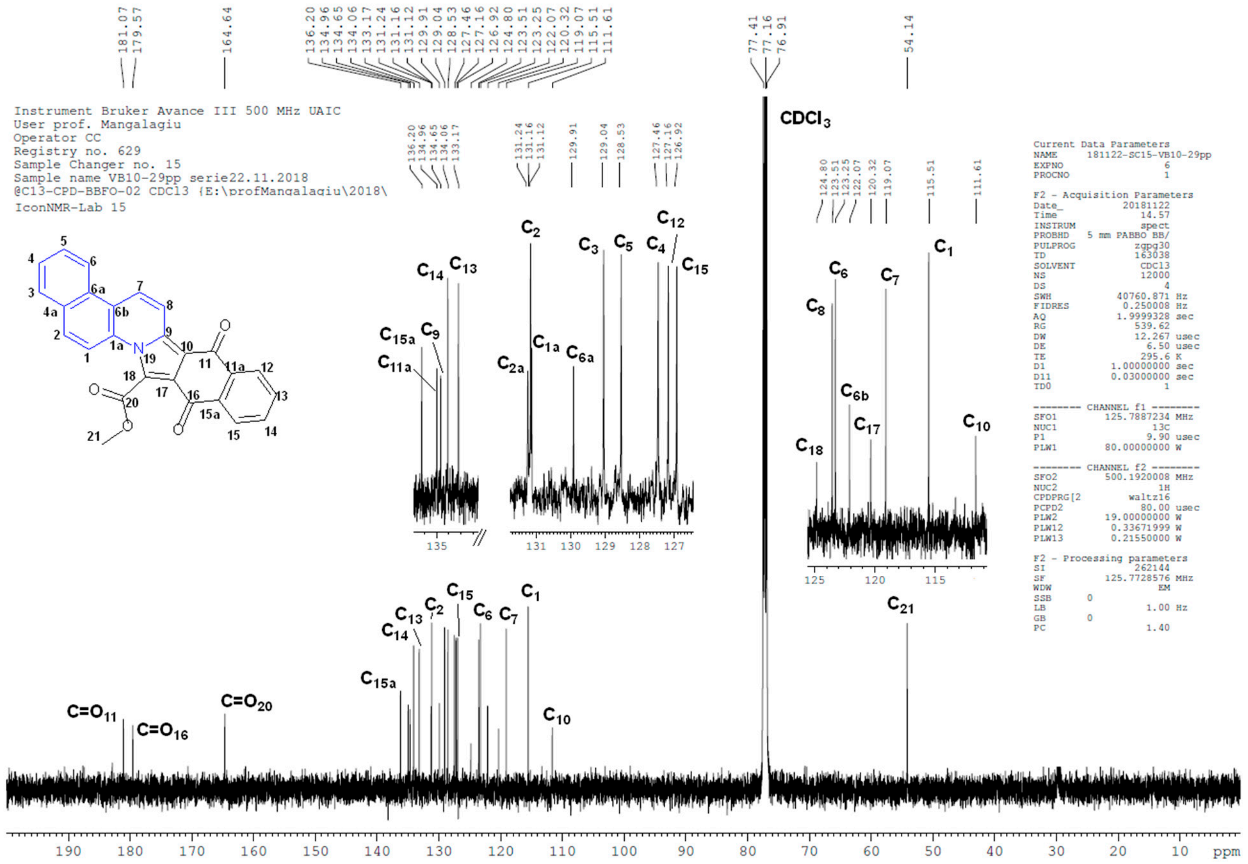

Figure S6. ^{13}C–NMR spectrum of methyl 9,14–dioxo–9,14–dihydrobenzo[f]benzo[5,6]isoindolo[2,1–a]quinoline–15–carboxylate (**7b**).

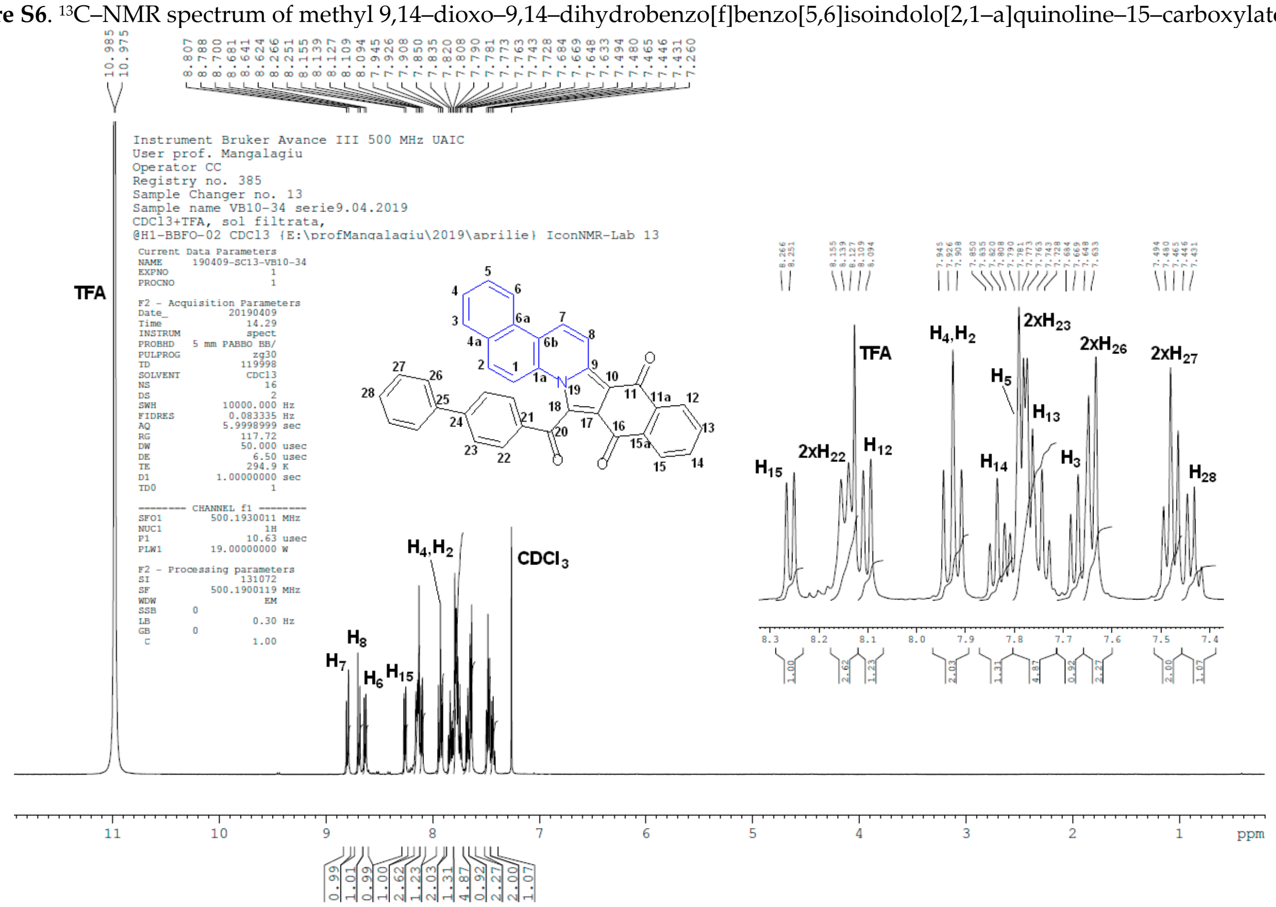

Figure S7. ^{1}H–NMR spectrum of15–([1,1'–biphenyl]–4–carbonyl)benzo[f]benzo[5,6]isoindolo[2,1–a]quinoline–9,14–dione (**7d**).

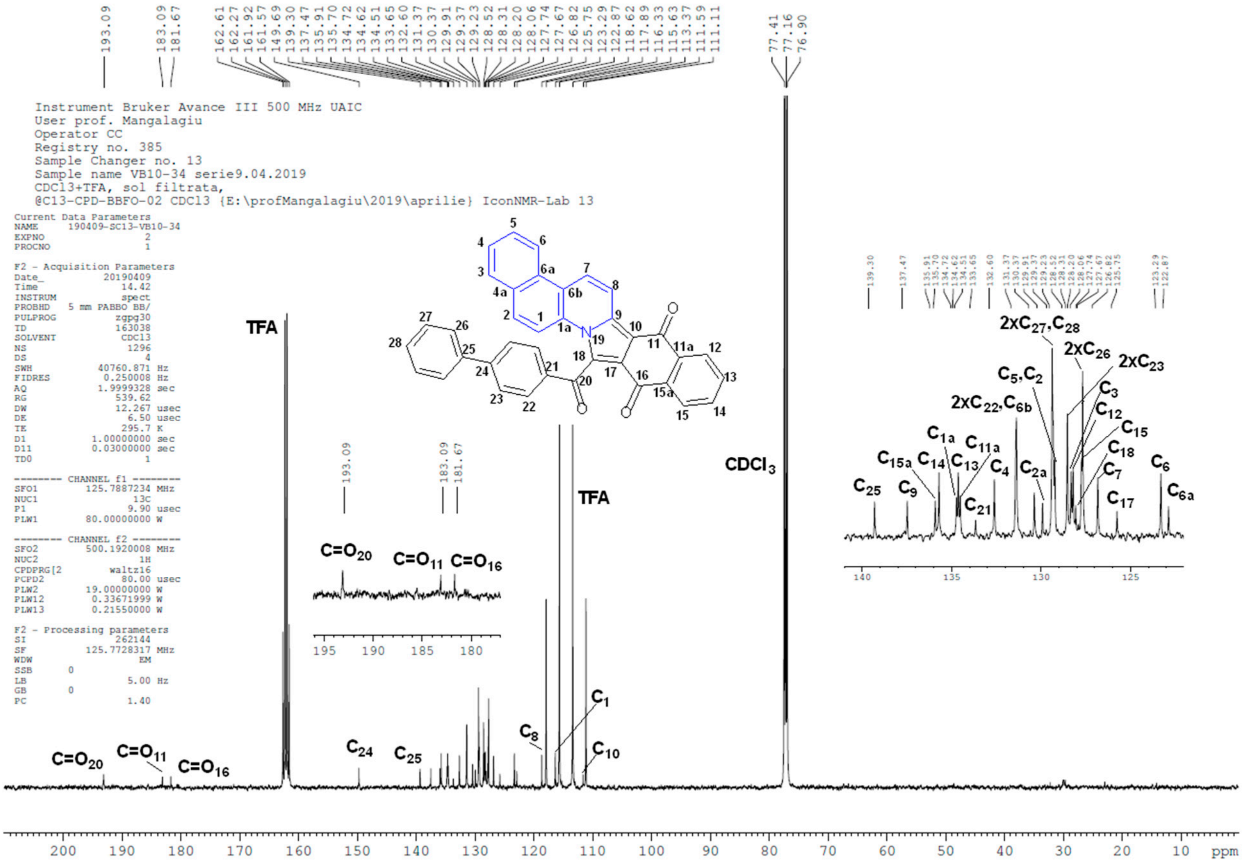

Figure S8. ^{13}C–NMR spectrum of15–([1,1'–biphenyl]–4–carbonyl)benzo[f]benzo[5,6]isoindolo[2,1–a]quinoline–9,14–dione (**7d**).

2. Anticancer results of all tested compounds **HybB[f]Q 6a–f** and **7a–e**

Table S1. Percentage growth inhibition (PGI %, μM)[a]of salts **3a–g** against the NCI 60 human cancer cell lines in the single–dose assay.

Cell type	Salts / Growth inhibition percent (PGI%)[a]						
	3a	3b	3c	3d	3e	3f	3g
Leukemia							
CCRF–CEM	0	3	4	72	48	68	41
HL–60 (TB)	0	20	0	**99**	78	**100**(62)[b]	70
K–562	0	34	5	**92**	**84**	**100**(26)[b]	**83**
MOLT–4	0	33	0	**86**	68	**100**(3)[b]	67
RPMI–8226	0	40	9	**95**	75	**93**	67
SR	4	40	15	**98**	66	**100**(28)[b]	**88**
Non-small Cell Lung Cancer							
A549/ATCC	0	9	3	76	62	59	43
EKVX	11	10	12	71	57	59	43
HOP–62	0	0	4	64	56	56	28
HOP–92	0	15	0	**100**(8)[b]	**88**	**93**	**87**
NCI–H226	9	22	19	64	41	-	32
NCI–H23	2	29	3	73	72	56	41
NCI–H322M	0	5	0	57	31	36	37

NCI–460	0	0	0	**96**	**81**	73	46
NCI–H522	0	11	4	78	71	64	25
Colon Cancer							
COLO 205	0	7	0	**94**	74	75	53
HCC–2998	1	0	0	**83**	52	**88**	54
HCT–116	0	48	1	**88**	70	**85**	**81**
HCT–15	0	0	0	43	13	47	23
HT29	0	0	0	**88**	**80**	**87**	79
KM12	0	15	5	74	65	72	23
SW–620	0	3	0	**81**	64	72	51
CNS Cancer							
SF–268	0	26	18	57	62	52	16
SF–295	0	5	0	77	70	66	53
SF–539	1	23	9	75	47	39	65
SNB–19	0	32	24	74	68	74	44
SNB–75	3	13	2	57	18	29	2
U251	3	40	28	**82**	72	70	46
Melanoma							
LOX IMVI	5	9	4	**100**(24)[b]	54	**89**	**82**
MALME–3M	6	32	4	**87**	66	57	35
M14	0	21	1	**83**	62	76	39
MDA–MB–435	0	33	0	**87**	63	61	38
SK–MEL–2	0	0	0	64	44	37	13
SK–MEL–28	0	6	0	56	33	26	21
SK–MEL–5	0	63	21	**100**(89)[b]	**100**(32)[b]	**100**(35)[b]	62
UACC–257	0	8	0	**95**	**85**	47	48
UACC–62	1	43	8	68	44	34	36
Ovarian Cancer							
IGROV1	0	14	0	69	33	52	51
OVCAR–3	0	36	0	70	48	69	37
OVCAR–4	0	39	4	**80**	67	**91**	37
OVCAR–5	0	0	3	69	42	42	47
OVCAR–8	0	1	0	78	74	74	49
NCI/ADR–RES	0	0	0	19	2	14	13
SK–OV–3	0	12	2	66	50	47	27
Renal Cancer							
786–0	0	13	2	66	33	46	48
A498	0	0	0	25	0	0	0
ACHN	0	0	0	50	21	44	33
CAKI–1	10	12	4	47	21	67	27
RXF 393	1	0	0	**94**	44	68	46
SN12C	0	15	6	**85**	**83**	74	46

TK–10	0	0	0	59	27	31	35
UO–31	21	16	16	70	44	71	68
Prostate Cancer							
PC–3	9	29	2	**81**	73	86	64
DU–145	0	0	0	64	44	58	32
Breast Cancer							
MCF7	4	39	13	**88**	**82**	**90**	61
MDA–MB–231/ATCC	0	21	0	76	60	58	48
HS 578T	0	12	1	73	41	56	32
BT–549	0	0	0	79	67	44	32
T–47D	0	10	3	56	62	64	35
MDA–MB–468	17	**85**	**76**	**100**(10)[b]	**97**	**100**(10)[b]	69

[a]The number reported for the one-dose assay, percentage growth inhibition (PGI), is growth relative to the no-drug control, and relative to the time zero number of cells; [b] Cytotoxic effect; lethality percent is represented in brackets; the most active compounds are highlighted in bold and red.

Table S2. Percentage growth inhibition (PGI %, μM)[a]of cycloadducts **6a–f** and **7a–e** against the NCI 60 human cancer cell lines in the single–dose assay.

**Cell type**	**Cycloadducts / Growth inhibition percent (PGI%)[a]**										
	**6a**	**6b**	**6c**	**6d**	**6e**	**6f**	**7a**	**7b**	**7c**	**7d**	**7e**
Leukemia											
CCRF–CEM	26	0	0	0	0	0	0	2	0	0	0
HL–60 (TB)	0	0	0	0	3	0	0	1	0	0	0
K–562	17	0	0	0	0	0	0	0	0	0	0
MOLT–4	10	0	0	3	0	0	0	0	0	0	0
RPMI–8226	33	0	0	13	0	2	0	0	0	0	0
SR	28	24	5	20	20	5	4	9	6	0	0
Non-small Cell Lung Cancer											
A549/ATCC	0	0	3	0	0	0	0	0	0	7	0
EKVX	8	0	3	20	0	0	3	3	0	0	3
HOP–62	0	0	0	20	0	7	0	8	0	1	8
HOP–92	25	0	3	17	0	0	0	0	0	0	1
NCI–H226	32	0	0	22	13	20	0	8	0	-	0
NCI–H23	26	0	0	17	0	0	5	7	3	2	4
NCI–H322M	25	0	0	0	0	0	0	1	2	0	0
NCI–460	43	0	0	0	0	0	0	0	0	0	0
NCI–H522	6	0	0	1	1	0	0	4	0	0	7
Colon Cancer											
COLO 205	19	0	0	1	0	0	0	0	0	0	0
HCC–2998	0	0	0	0	0	0	0	0	0	4	0
HCT–116	19	0	2	11	0	8	0	0	0	0	0
HCT–15	23	0	2	0	0	0	0	0	0	0	0

HT29	5	0	0	1	0	0	0	0	0	0	0
KM12	8	0	0	1	0	0	0	1	0	0	0
SW–620	11	0	0	8	0	0	0	1	0	0	0
CNS Cancer											
SF–268	11	0	0	0	0	0	0	0	0	0	0
SF–295	15	0	7	16	1	8	0	3	0	0	1
SF–539	30	0	1	3	0	8	2	9	0	0	12
SNB–19	17	0	19	11	0	5	1	10	0	0	4
SNB–75	29	0	4	20	0	3	0	4	0	0	13
U251	2	0	3	8	1	0	1	0	0	1	0
Melanoma											
LOX IMVI	25	0	0	9	0	6	5	5	2	0	10
MALME–3M	26	0	4	5	6	0	3	12	0	0	0
M14	12	0	0	0	0	0	0	6	0	4	0
MDA–MB–435	7	0	1	1	0	0	0	0	0	0	0
SK–MEL–2	0	0	0	0	0	0	0	0	0	0	0
SK–MEL–28	10	0	0	0	0	0	0	0	0	0	0
SK–MEL–5	29	0	2	0	0	0	0	0	0	0	0
UACC–257	0	0	0	0	0	0	0	1	0	10	0
UACC–62	7	0	6	20	17	10	0	8	0	2	3
Ovarian Cancer											
IGROV1	24	0	0	19	0	1	0	0	14	0	0
OVCAR–3	5	0	24	0	0	9	0	0	0	0	0
OVCAR–4	36	0	0	34	2	20	0	0	0	0	0
OVCAR–5	5	0	0	1	0	0	0	0	0	0	0
OVCAR–8	26	0	0	4	0	0	0	0	0	0	0
NCI/ADR–RES	21	1	0	8	0	4	1	2	0	0	0
SK–OV–3	0	0	0	10	0	12	0	7	0	0	5
Renal Cancer											
786–0	11	0	0	4	4	8	0	0	0	0	0
A498	0	0	0	0	0	0	0	0	0	0	0
ACHN	28	0	0	41	0	17	0	0	8	0	18
CAKI–1	9	0	0	38	0	15	5	9	11	4	8
RXF 393	22	5	25	0	0	1	0	8	0	1	-
SN12C	17	0	4	22	0	0	1	0	0	4	0
TK–10	0	0	0	0	3	0	0	0	0	0	0
UO–31	37	8	12	45	21	33	18	26	26	10	30
Prostate Cancer											
PC–3	23	0	1	19	5	3	9	0	11	0	5
DU–145	26	0	0	0	0	0	0	0	0	0	0
Breast Cancer											
MCF7	36	7	15	10	8	10	23	14	10	0	7

MDA–MB–231/ATCC	42	0	0	11	0	1	6	4	5	0	10
HS 578T	9	0	7	19	0	6	0	2	0	1	7
BT–549	30	0	0	0	0	0	0	0	0	0	0
T–47D	21	0	6	3	19	1	0	10	0	7	0
MDA–MB–468	29	0	10	0	7	0	2	2	0	5	0

[a]The number reported for the one-dose assay, percentage growth inhibition (PGI), is growth relative to the no-drug control, and relative to the time zero number of cells.

3. CheckCIF files for X–ray data of compounds **6c** and **7c**

CheckCIF files for X–ray data of trimethyl benzo[f]pyrrolo[1,2–a]quinoline–1,2,3–tricarboxylate (**6c**).

checkCIF/PLATON report

Structure factors have been supplied for datablock(s) anto001tr

THIS REPORT IS FOR GUIDANCE ONLY. IF USED AS PART OF A REVIEW PROCEDURE FOR PUBLICATION, IT SHOULD NOT REPLACE THE EXPERTISE OF AN EXPERIENCED CRYSTALLOGRAPHIC REFEREE.

No syntax errors found. CIF dictionary Interpreting this report

Datablock: anto001tr

```
Bond precision:   C-C = 0.0028 A          Wavelength=1.54184

Cell:          a=7.7700(6)       b=8.0545(5)       c=15.5105(11)
               alpha=102.774(5)  beta=99.140(6)    gamma=103.718(6)
Temperature:   293 K

                Calculated                  Reported
Volume          896.51(12)                  896.51(11)
Space group     P -1                        P -1
Hall group      -P 1                        -P 1
Moiety formula  C22 H17 N O6                C22 H17 N O6
Sum formula     C22 H17 N O6                C22 H17 N O6
Mr              391.37                      391.36
Dx,g cm-3       1.450                       1.450
Z               2                           2
Mu (mm-1)       0.889                       0.889
F000            408.0                       408.0
F000'           409.39
h,k,lmax        9,9,18                      9,9,18
Nref            3456                        3388
Tmin,Tmax       0.958,0.965                 0.760,1.000
Tmin'           0.752

Correction method= # Reported T Limits: Tmin=0.760 Tmax=1.000
AbsCorr = GAUSSIAN

Data completeness= 0.980            Theta(max)= 70.906

R(reflections)= 0.0438( 2583)                  wR2(reflections)=
                                               0.1266( 3388)
S = 1.032                 Npar= 265
```

```
The following ALERTS were generated. Each ALERT has the format
       test-name_ALERT_alert-type_alert-level.
Click on the hyperlinks for more details of the test.

Alert level C
PLAT911_ALERT_3_C Missing FCF Refl Between Thmin & STh/L=    0.600          2 Report

Alert level G
PLAT199_ALERT_1_G Reported _cell_measurement_temperature ..... (K)        293 Check
PLAT200_ALERT_1_G Reported   _diffrn_ambient_temperature ..... (K)        293 Check
PLAT912_ALERT_4_G Missing # of FCF Reflections Above STh/L=  0.600         66 Note
PLAT941_ALERT_3_G Average HKL Measurement Multiplicity ...........        1.9 Low
PLAT978_ALERT_2_G Number C-C Bonds with Positive Residual Density.          2 Info

   0 ALERT level A = Most likely a serious problem - resolve or explain
   0 ALERT level B = A potentially serious problem, consider carefully
   1 ALERT level C = Check. Ensure it is not caused by an omission or oversight
   5 ALERT level G = General information/check it is not something unexpected

   2 ALERT type 1 CIF construction/syntax error, inconsistent or missing data
   1 ALERT type 2 Indicator that the structure model may be wrong or deficient
   2 ALERT type 3 Indicator that the structure quality may be low
   1 ALERT type 4 Improvement, methodology, query or suggestion
   0 ALERT type 5 Informative message, check
```

It is advisable to attempt to resolve as many as possible of the alerts in all categories. Often the minor alerts point to easily fixed oversights, errors and omissions in your CIF or refinement strategy, so attention to these fine details can be worthwhile. In order to resolve some of the more serious problems it may be necessary to carry out additional measurements or structure refinements. However, the purpose of your study may justify the reported deviations and the more serious of these should normally be commented upon in the discussion or experimental section of a paper or in the "special_details" fields of the CIF. checkCIF was carefully designed to identify outliers and unusual parameters, but every test has its limitations and alerts that are not important in a particular case may appear. Conversely, the absence of alerts does not guarantee there are no aspects of the results needing attention. It is up to the individual to critically assess their own results and, if necessary, seek expert advice.

Publication of your CIF in IUCr journals

A basic structural check has been run on your CIF. These basic checks will be run on all CIFs submitted for publication in IUCr journals (*Acta Crystallographica*, *Journal of Applied Crystallography*, *Journal of Synchrotron Radiation*); however, if you intend to submit to *Acta Crystallographica Section C* or *E* or *IUCrData*, you should make sure that full publication checks are run on the final version of your CIF prior to submission.

Publication of your CIF in other journals

Please refer to the *Notes for Authors* of the relevant journal for any special instructions relating to CIF submission.

PLATON version of 12/09/2022; check.def file version of 09/08/2022

Datablock anto001tr - ellipsoid plot

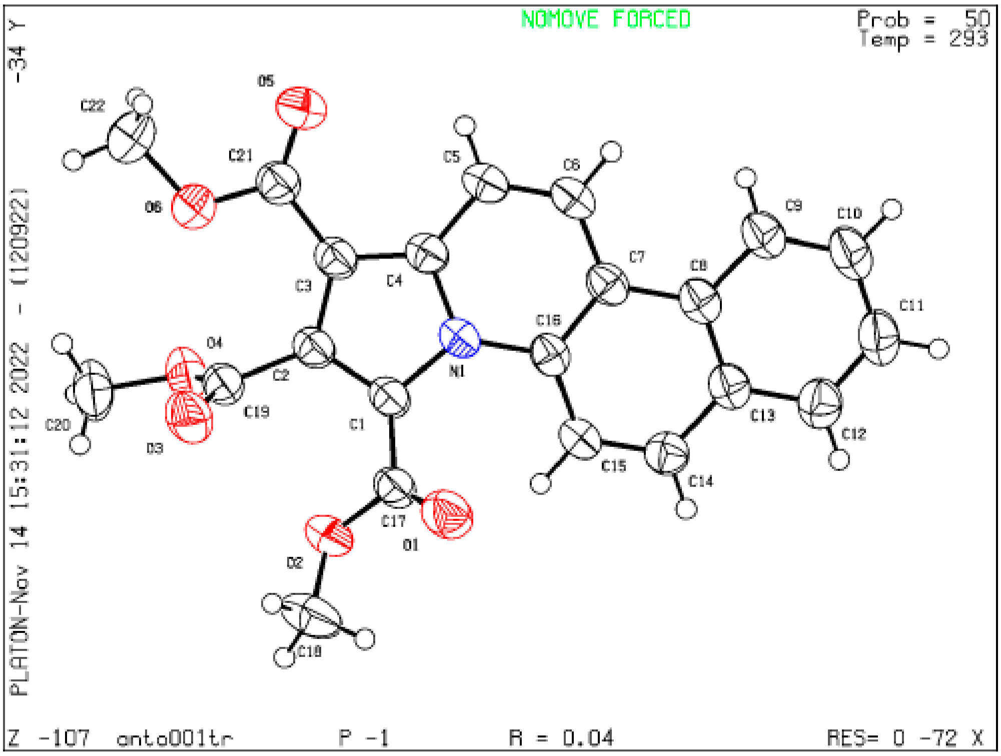

CheckCIF files for X–ray data ofethyl 9,14–dioxo–9,14–dihydrobenzo[f]benzo[5,6]isoindolo[2,1–a]quinoline–15–carboxylate (**7c**).

checkCIF/PLATON report

Structure factors have been supplied for datablock(s) anto002tr

THIS REPORT IS FOR GUIDANCE ONLY. IF USED AS PART OF A REVIEW PROCEDURE FOR PUBLICATION, IT SHOULD NOT REPLACE THE EXPERTISE OF AN EXPERIENCED CRYSTALLOGRAPHIC REFEREE.

No syntax errors found. CIF dictionary Interpreting this report

Datablock: anto002tr

```
Bond precision:   C-C = 0.0030 A          Wavelength=1.54184

Cell:             a=6.9814(4)        b=11.3238(8)      c=13.1202(9)
                  alpha=78.690(6)    beta=75.608(6)    gamma=81.707(5)
Temperature:      293 K

                  Calculated                   Reported
Volume            980.18(12)                   980.18(12)
Space group       P -1                         P -1
Hall group        -P 1                         -P 1
Moiety formula    C27 H17 N O4                 C27 H17 N O4
Sum formula       C27 H17 N O4                 C27 H17 N O4
Mr                419.42                       419.42
Dx,g cm-3         1.421                        1.421
Z                 2                            2
Mu (mm-1)         0.781                        0.781
F000              436.0                        436.0
F000'             437.37
h,k,lmax          8,13,16                      8,13,16
Nref              3770                         3685
Tmin,Tmax         0.945,0.969                  0.734,1.000
Tmin'             0.544

Correction method= # Reported T Limits: Tmin=0.734 Tmax=1.000
AbsCorr = GAUSSIAN

Data completeness= 0.977            Theta(max)= 70.862

R(reflections)= 0.0453( 2678)                         wR2(reflections)=
                                                      0.1353( 3685)
S = 1.029                  Npar= 290
```

```
The following ALERTS were generated. Each ALERT has the format
       test-name_ALERT_alert-type_alert-level.
Click on the hyperlinks for more details of the test.
```

Alert level C

```
PLAT220_ALERT_2_C NonSolvent   Resd 1  C   Ueq(max)/Ueq(min) Range        3.8 Ratio
PLAT222_ALERT_3_C NonSolvent Resd 1  H   Uiso(max)/Uiso(min) Range        4.1 Ratio
PLAT242_ALERT_2_C Low    'MainMol' Ueq as Compared to Neighbors of        C26 Check
PLAT360_ALERT_2_C Short  C(sp3)-C(sp3) Bond  C26      - C27      .       1.38 Ang.
PLAT911_ALERT_3_C Missing FCF Refl Between Thmin & STh/L=    0.600          6 Report
```

Alert level G

```
PLAT003_ALERT_2_G Number of Uiso or Uij Restrained non-H Atoms ...          1 Report
PLAT063_ALERT_4_G Crystal Size Possibly too Large for Beam Size ..       0.78 mm
PLAT186_ALERT_4_G The CIF-Embedded .res File Contains ISOR Records          1 Report
PLAT199_ALERT_1_G Reported _cell_measurement_temperature ..... (K)        293 Check
PLAT200_ALERT_1_G Reported   _diffrn_ambient_temperature ..... (K)        293 Check
PLAT333_ALERT_2_G Large Aver C6-Ring C-C Dist C2       -C11       .      1.44 Ang.
PLAT860_ALERT_3_G Number of Least-Squares Restraints .............          6 Note
PLAT912_ALERT_4_G Missing # of FCF Reflections Above STh/L=  0.600         80 Note
PLAT941_ALERT_3_G Average HKL Measurement Multiplicity ...........        2.0 Low
PLAT978_ALERT_2_G Number C-C Bonds with Positive Residual Density.          0 Info
```

```
   0 ALERT level A = Most likely a serious problem - resolve or explain
   0 ALERT level B = A potentially serious problem, consider carefully
   5 ALERT level C = Check. Ensure it is not caused by an omission or oversight
  10 ALERT level G = General information/check it is not something unexpected

   2 ALERT type 1 CIF construction/syntax error, inconsistent or missing data
   6 ALERT type 2 Indicator that the structure model may be wrong or deficient
   4 ALERT type 3 Indicator that the structure quality may be low
   3 ALERT type 4 Improvement, methodology, query or suggestion
   0 ALERT type 5 Informative message, check
```

It is advisable to attempt to resolve as many as possible of the alerts in all categories. Often the minor alerts point to easily fixed oversights, errors and omissions in your CIF or refinement strategy, so attention to these fine details can be worthwhile. In order to resolve some of the more serious problems it may be necessary to carry out additional measurements or structure refinements. However, the purpose of your study may justify the reported deviations and the more serious of these should normally be commented upon in the discussion or experimental section of a paper or in the "special_details" fields of the CIF. checkCIF was carefully designed to identify outliers and unusual parameters, but every test has its limitations and alerts that are not important in a particular case may appear. Conversely, the absence of alerts does not guarantee there are no aspects of the results needing attention. It is up to the individual to critically assess their own results and, if necessary, seek expert advice.

Publication of your CIF in IUCr journals

A basic structural check has been run on your CIF. These basic checks will be run on all CIFs submitted for publication in IUCr journals (*Acta Crystallographica*, *Journal of Applied Crystallography*, *Journal of Synchrotron Radiation*); however, if you intend to submit to *Acta Crystallographica Section C* or *E* or *IUCrData*, you should make sure that full publication checks are run on the final version of your CIF prior to submission.

Publication of your CIF in other journals

Please refer to the *Notes for Authors* of the relevant journal for any special instructions relating to CIF submission.

PLATON version of 12/09/2022; check.def file version of 09/08/2022

Datablock anto002tr - ellipsoid plot

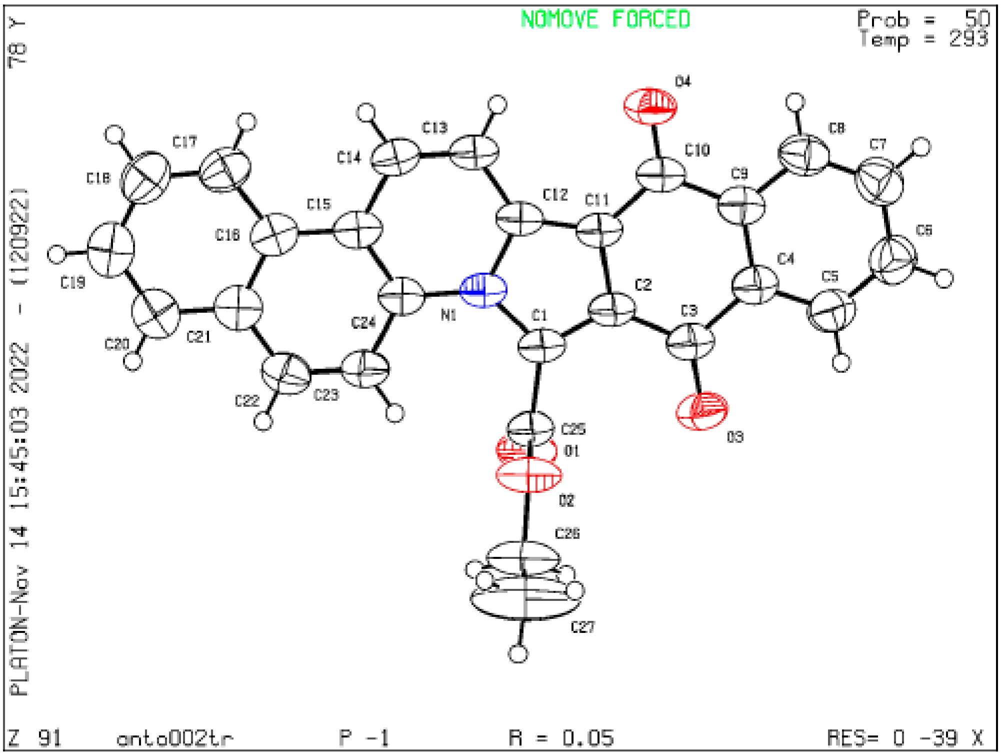

Supplement: Supplementary file 1 [file ijms-24-08124-s001.zip › ijms-2329778-supplementary.pdf]
